# Primary care provider's job satisfaction and organizational commitment after COVID-19 restrictions ended: A mixed-method study using a mediation model

**DOI:** 10.3389/fpsyg.2022.873770

**Published:** 2022-10-13

**Authors:** Quan Wang, Xinyu Liu, Ting Wang, Zemeng Zhu, Li Yang, Shasha Guo, Hui Li, Qiang Sun

**Affiliations:** ^1^Center for Health Management and Policy Research, School of Public Health, Cheeloo College of Medicine, Shandong University, Jinan, China; ^2^National Health Commission (NHC) Key Lab of Health Economics and Policy Research, Shandong University, Jinan, China; ^3^Institute of Health Policy, Management and Evaluation (IHPME), Dalla Lana School of Public Health, University of Toronto, Toronto, ON, Canada; ^4^Jinan Municipal Center for Disease Control and Prevention, Jinan, Shandong Province, China; ^5^School of Public Health, Peking University, Beijing, China; ^6^School of Basic Medicine Science, Shandong University, Jinan, China; ^7^School of Integrated Traditional Chinese and Western Medicine, Binzhou Medical University, Yantai, China

**Keywords:** primary care provider, job satisfaction, organizational commitment, COVID-19, mixed method, mediation model

## Abstract

**Objectives:**

More and more countries have decided to cancel most or even all COVD-19 restrictions. However, it is unclear how ending of restrictions will affect primary care providers' job satisfaction and organizational commitment. Our objectives are to explore the current status and possible change in primary care providers' job satisfaction and organizational commitment after massive restriction policies ended in China.

**Methods:**

This was a mixed-method study that utilized structured questionnaires and semi-structured qualitative individual interviews. The 20-item Minnesota Satisfaction Questionnaire (MSQ) and 25-item organizational commitment survey were adopted to assess job satisfaction and organization commitment. Descriptive statistics and mediation models, as well as inductive thematic analysis, were used to analyze quantitative and qualitative data.

**Results:**

A total of 18 interviews and 435 valid survey responses were included in our analysis. The average scores for job satisfaction and organizational commitment were 80.6 and 90.8. The thematic analysis revealed one major theme: ethical and moral responsibility to provide care as primary care providers, on which we established a mediation model. The mediation analysis revealed that normative commitment could positively affect the other four dimensions of organizational commitment and job satisfaction. The direct effect of affective commitment on job satisfaction was significant (LLCI = 0.11, ULCI = 0.31), and the mediators were identified to have a partial mediating effect instead of a total mediating effect.

**Conclusion:**

After COVID-19 restrictions end, the job satisfaction and organizational commitment of primary care providers will return to levels before the pandemic and during this estimated process, a brief rise in resignation is predictable. The normative commitment positively affects the other four dimensions of organizational commitment and job satisfaction for primary care providers, which suggests a possible way to motivate primary care providers when restrictions end.

## Introduction

At the end of 2019, a new highly infectious respiratory virus, named severe acute respiratory syndrome coronavirus 2 (SARS-CoV-2), attacked the world and posed a significant challenge to all countries (The Lancet, 2020; World Health Organization, 2020). In response to the pandemic, China's government implemented a series of restriction policies in February 2020 to control the spread of SARS-CoV-2 (Wu and McGoogan, 2020). These restrictions included a national lockdown policy, active case surveillance (monitoring of floating population, massive monitoring of body temperatures, epidemiological investigation, trace track of patients with COVID-19, suspected patients, and close contacts), massive quarantine and management, mandatory face mask, prohibition on all forms of gathering, meeting and school activities, travel ban, and traffic restrictions (Ding and Zhang, 2022). By implementing these restriction policies, China successfully controlled COVID-19 in a few months (Chen H. et al., 2021). Since 26 April 2020, the very last patients with COVID-19 in Wuhan got fully recovered and discharged from the hospital, and the incremental COVID-19 cases in China were under 20 per day. Therefore, regions and provinces in China began to cancel the state of emergency and social restrictions gradually, including: lockdown policy, mandatory face masks, travel ban, prohibition on all forms of gathering, meeting and school activities, and traffic restrictions (Xu et al., 2020). Although some restriction policies remained, such as case surveillance and health code (a type of QR code for everyone to prove health status), they had little impact on people's lives insignificantly (Jin et al., 2022). Schools, factories, malls, and all other public facilitates reopened until June 2020, people could freely travel to other provinces, and the society basically returned to normal. The restrictions impacted both primary care providers and patients. Due to the lockdown policy and complicated admission procedure, many patients failed to access the healthcare services they needed during the restriction period (Xiang et al., 2020; Chen X. et al., 2021). While the burden on primary care providers' shoulders was not released, because there were many extra works, including: (1) identifying the residents with fever and providing basic healthcare services; (2) managing and quarantining the floating population; (3) health education and campaigns; and (4) help local governments and CDCs to control the spread of SARS-CoV-2 (Fu et al., 2020; Nation Health Commission, 2020; Pan et al., 2020; Wuhan Government, 2020; Xiong et al., 2020). Primary care providers are employees working in primary care institutes and provide primary health services to local residents, like prevention, rehabilitation, diagnosis and treatment of common and frequently occurring diseases, and health education (Liu et al., 2021). Some of them may also undertake administrative works, since specialized administrators and managers are very rare in Chinese primary care institute (Hao et al., 2022). These added responsibilities have placed primary care providers at relatively dangerous risk of COVID-19 exposure (Lai et al., 2020).

Healthcare workers exposed to COVID-19 are at high risk of developing mental health. A Chinese-based study of 1257 healthcare providers, conducted from January to February 2020, found that a considerable proportion of participants reported suffering from symptoms of depression, anxiety, insomnia, and distress (Lai et al., 2020). Zhang and his colleagues surveyed 450 healthcare providers in urban primary care institutions in February 2020 and concluded that experiencing psychological distress was a common phenomenon among study participants (Zhang et al., 2021). Aymerich synthesized current evidence and concluded that 33% of healthcare workers exposed to COVID-19 reported depressive symptoms, 42% anxiety features, 40% acute stress, 32% post-traumatic symptoms, 42% insomnia, and 37% burnout (Aymerich et al., 2022). Muller tried to explore the risk factors associated with mental health problems in healthcare workers and found that exposure to patients with COVID-19, being a woman, and worrying about being infected were the most common ones (Muller et al., 2020). In addition, the shortage of protective equipment (Lu et al., 2020), the environment of isolation (Zhang et al., 2020), and financial threats (Bohlken et al., 2020) were also important risk factors. Previous studies have proved that job-related stress, high workload, and unhealthy work environment could lead to job satisfaction deterioration among medical staff (Alrawashdeh et al., 2021). However, according to Yu, the job satisfaction of frontline medical staff during the restriction period remained decent, even higher than the previous similar measures among medical staff (Yu et al., 2020). Nevertheless, the status quo of care providers after restriction policies ended is still not well-known, especially how they feel about their job and working institutes.

As estimated, with high vaccination rates and the milder Omicron variant, the end of the pandemic is near (Murray, 2022; World Health Organization, 2022). Actually, many countries have canceled most or even all COVID-19 restrictions, like the UK, Sweden, and Denmark. In foreseeable future, more and more will join them. Therefore, understanding the change in medical care providers' work attitudes after restriction policies end is rather important, because work attitudes can highly influence the job performance and function of the entire organization (Wang, 2008).

Job satisfaction is an individual's psychological feeling composed of attitude, belief, emotion, and evaluation of his or her work, which is related to work performance and patient quality of care (Noroxe et al., 2019). Organizational commitment encompasses a series of behaviors performed by employees that lead them to undertake efforts for the good of the institution, a yearning to remain in it, and accepting its goals and values (Porter and Lawler, 1965). Its core element is whether employees are willing to believe, commit to, and stay in the organization (Lu et al., 2019), which has a significant predictive effect on employee turnover and retention (Ling et al., 2001; Wagner, 2007). Organizational commitment has shown to have a significant positive correlation with job satisfaction (Baek et al., 2019; Gonzalez-Gancedo et al., 2019). In the present study, we first assessed the job satisfaction and organizational commitment of primary care workers 2 months after most COVID-19 restrictions ended in China. Then we tried to explore how organizational commitment impacted job satisfaction. The results of this study could provide evidence for professionals to facilitate further motivation plans for primary care providers and maintain the reliance on the primary care system, which is essential for family and community health (Rasanathan and Evans, 2020).

## Methods

### Study design

Due to complex interactions between job satisfaction and organizational commitment, and in order to gain a comprehensive and in-depth overview both narratively and numerically, a mixed-method design was implemented. In the current study, the research aim was addressed using both qualitative and quantitative methods thus, achieving a comprehensive interpretation of data based on the power of methods triangulation (Creswell and Clark, 2017). A mixed-method approach is characterized by the integration that occurs between qualitative and quantitative methods at single or multiple steps of research (Moseholm and Fetters, 2017). In our study, qualitative and quantitative methods were implemented simultaneously.

### Study setting and population

For the quantitative methods, we distributed a web-based questionnaire to all (*n* = 989) primary care providers in District H of Jinan. Compared with other regions in China, the restriction policies in District H were not special, including lockdown policy, active case surveillance, traffic restrictions, etc. The lockdown policy was canceled on 17 February 2020 and most public facilities reopened at 1st April of the same year. Since then, there were no patients with COVID-19 in District H for more than 1 year. Therefore, the social restrictions in District H were quite limited, as mentioned above. Just as in other regions of China, District H has a highly hospital-centered healthcare system. Although the primary care institute is easy to access and get primary care, a significant part of residents would like to choose the hospital due to various reasons (so as the other part of China). Based on the population and socioeconomic status of Jinan city, we selected three districts in Jinan (Lixia, Huaiyin, and Changqing districts) to conduct our qualitative study. From each district, we randomly selected two Community Health Centers (CHCs) and conducted a face-to-face interview with the primary care providers and managers. In every CHC selected, two primary care providers and one manager (usually the director) were interviewed in appropriate and accessible meeting rooms.

### Instruments and validity

#### Measurement of job satisfaction

The job satisfaction of primary care providers was measured by The Minnesota Satisfaction Questionnaire (MSQ) short form validated in Chinese which contained 20 questions (University of Minnesota, 1977). The short form uses Likert ratings (1 to 5 from very dissatisfied to very satisfied). A higher score indicated a better job satisfaction. The Chinese version of the MSQ short form has been widely used in various studies about health workers, and its Cronbach's alpha ranged from 0.88 to 0.93 (Ge et al., 2011; Liu et al., 2018; Zhou et al., 2019). The MSQ short form assesses two aspects of job satisfaction: intrinsic job satisfaction and extrinsic job satisfaction. Intrinsic job satisfaction refers to whether people feel satisfied with the factors related to the nature of their jobs; whereas extrinsic job satisfaction refers to whether people feel satisfied with the factors related to the working conditions that are external to their jobs (Spector, 1997).

#### Measurement of organizational commitment

The questionnaire on Chinese employees' organizational commitment, which Ling develops in 2001 (Ling et al., 2001), was used and validated to measure the organizational commitment of medical workers by Gao in 2013 (Gao, 2014). The organizational commitment was assessed by a model composed of five dimensions: affective, normative, ideal, economic, and choice commitment. Affective commitment is defined as the solid emotional attachment employee has for the organization, and is associated with devotion in support of the organization regardless of reward and firm intention to maintain membership in resistance to any lure. Normative commitment is a sense of obligation and responsibility to exert effort for the organization, with the guidance of social norms and professional ethics. Ideal commitment is the wishes of the potential opportunity organization for improvement and promotion, when personal growth, specialty utilization, and realization of ambition are valued by the employee. Choice commitment is the fear of uncertainty of leaving the organization and difficulty of re-employment due to existing shortages in expertise and practice. Finally, economic commitment is the awareness of financial income loss. Considering the unique characteristics of health workers, Gao adjusted the original questionnaire slightly and retested its validity. The Cronbach's alpha of the adjusted scale ranged from 0.78 to 0.86 (Gao, 2014). The scale contains 25 Likert (1 to 5 from very dissatisfied to very satisfied) questions that reflect the five dimensions (affective commitment, normative commitment, ideal commitment, economic commitment, and choice commitment) of organizational commitment. In other words, a higher score is indicative of a stronger organizational commitment.

### Data collection

The questionnaires were distributed to all primary care providers in Jinan's District H via Wenjuanxing, a webpage-based tool that allowed the participants to fill out the questionnaire on a computer or smart cellphone. Wenjuanxing allowed us to get results automatically. The period for data collection was 9 days, namely from 5 August 2020 to 13 August 2020 (~2 months after most COVID-19 restrictions ended).

The face-to-face semi-structured individual interview was applied as the qualitative data collection method. Participants could choose their interview date during the data collection period (from 5 August 2020 to 13 August 2020) according to their preference. Each interview lasted about 60 min and was conducted by trained interviewers using a semi-structured interview guideline. All interviews were audio-recorded and transcribed verbatim with participants' permission.

### Data analysis

Mixed methods were used in this study, including semi-structured interviews, which aimed to explore how five dimensions of organizational commitment affected job satisfaction qualitatively, and the mediation model aimed to quantitatively confirm the assumption we got from the interviews.

First, we collected and cleaned the data from Wenjuanxing. Any questionaries completed in 300 s were excluded from the analysis, to ensure high-quality responses. Second, descriptive statistics were conducted to examine the demographic data collected. Third, two researchers (QW and XL) individually analyzed the qualitative data gathered from the interview and explored the relationship between the five dimensions of organizational commitment and job satisfaction, based on which a hypothetical mediation model was established. Spearman coefficients were used to test the correlation between five dimensions of organizational commitment and job satisfaction. Then, a bootstrapped mediation analysis based on the PROCESS script was used to analyze the mediating effects of five dimensions of organization commitment to job satisfaction and test the hypothetical model. In order to control for any possible unequal distributions within clusters, the sociodemographic variables such as age, gender, working years, education, income, and professional title were included as predictors. All data were analyzed in SPSS 15.0.

## Results

A total of 528 primary care providers in District H took the survey with a response rate of 53.4%. After removing those submitted in <300 s or incorrectly filled, we are left with a final sample consisting of 435 valid responses.

The average age of respondents was 36 years old, and about 84.4% were female subjects. Concerning their career, only 20.0% of them got a budgeted post (a kind of permanent employment relation between individual and public institute) with an average of 7.76 years working in their current institution, about half of their total years of experience (14.1 years). About half of them (49.89%) had a master's degree, followed by those who had a bachelor degree (42.30%). Most people were engaged in only one type of professional field (73.33%), and most were public health professionals and nurses. The time they spent on patient care-related work far exceeded their time devoted to management work (72 vs. 20%). The average total scores for job satisfaction and organizational commitment were 80.60 and 90.75, respectively. For each dimension of organizational commitment, the average scores of emotional commitment, normative commitment, ideal commitment, economic commitment, and choice commitment were 19.10, 19.54, 19.15, 18.54, and 14.42, respectively (Table 1).

**Table 1:** Characteristics of included participants (*N* = 435).

**Discrete variables**	**Number (%)**
Gender	
Men	68 (15.63)
Women	367 (84.37)
Job status	
Budgeted post	87 (20.00)
Temporary contract labor	348 (80.00)
Professional qualification certificate	
No qualification certificate has been obtained yet	29 (6.67)
Qualification certificate of medical practitioner	100 (22.99)
Qualification certificate of licensed assistant physician	21 (4.83)
Village doctor's practice certificate	3 (0.69)
Other health technology qualification certificates	282 (64.83)
Technical title:	
Senior	1 (0.23)
Vice senior	18 (4.14)
Intermediate	99 (22.76)
Medical practitioner or occupation (assistant doctor)	89 (20.46)
Others	228 (52.41)
Most advanced degree	
Doctor	13 (2.99)
Master	217 (49.89)
Bachelor	184 (42.30)
Junior college	21 (4.83)
Senior high school and below	0 (0)
Professional field engaged	
Medical treatment (traditional Chinese medicine not included)	73 (17.87)
Traditional Chinese medicine	49 (11.26)
Public health services	174 (40.00)
Nursing	165 (37.93)
Others	110 (25.29)
Number of professional fields engaged	
1	319 (73.33)
2	98 (22.53)
3	17 (3.91)
5	1 (0.23)
Self-perceived income from medical practice in 2019, compared with 2018	
A significant improvement	17 (3.91)
An improvement	193 (44.37)
No change	188 (43.22)
A decline	30 (6.90)
A significant decline	7 (1.61)
Types of public medical insurance	
Medical insurance for urban and rural residents	37 (8.51)
Medical insurance for urban residents	311 (71.49)
No public medical insurance	87 (20.00)
Types of endowment insurance	
Basic endowment insurance for urban workers	412 (94.71)
Basic endowment insurance for urban and rural residents	12 (2.76)
No endowment insurance	11 (2.53)
**Continuous variables:**	**Mean (SE)**
Age (years)	36.20 ± 8.72
Working years (years)	14.12 ± 8.85
Working years in the current institute (years)	7.76 ± 8.20
Proportion of time occupied by management work (%)	20.45 ± 30.57
Proportion of time occupied by care service work (%)	72.36 ± 34.79
Annual income from medical practice in the 2019 (RMB)	48,400 ± 25,200
Working hours per week	41.73 ± 10.61
Job satisfaction	80.60 ± 13.80
Organizational commitment	90.75 ± 16.74
Affective commitment	19.10 ± 3.66
Normative commitment	19.54 ± 3.45
Ideal commitment	19.15 ± 4.08
Economic commitment	18.54 ± 3.93
Choice commitment	14.42 ± 4.59

**Figure 1:**
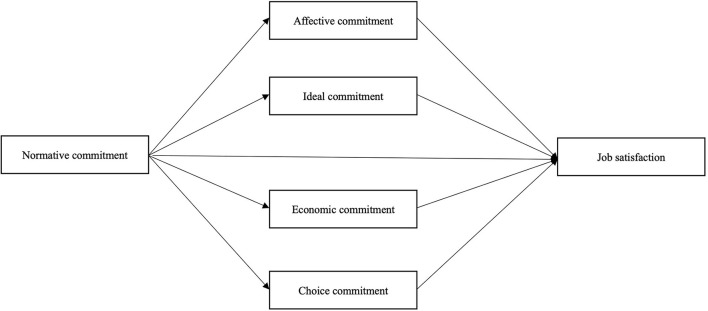
Hypothesized mediation model of organizational commitment and job satisfaction.

The interview results showed one major theme which is the ethical and moral responsibility of providing care. One participant summarized it as: “As a doctor, this is exactly the right time we need to fulfill our responsibilities to society. We are doing our best to get the work done.” Another participant said, “the sense of responsibility allows us to ignore personal gains and losses.” Therefore, we guessed the normative commitment shared effect on the other four dimensions of organizational commitment as well as job satisfaction. We proposed the relationship between organizational commitment and job satisfaction via a mediation model (Figure 1).

**Figure 2:**
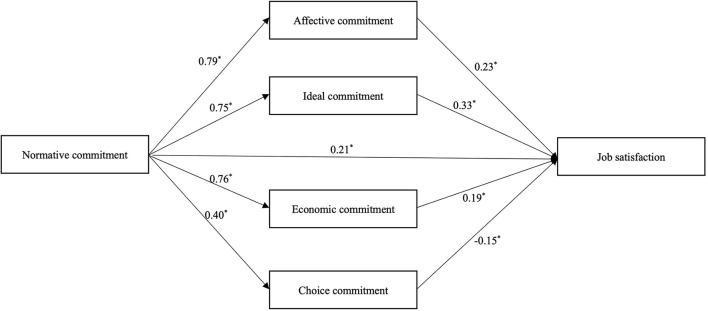
Mediation model of organizational commitment and job satisfaction. ^*^: *P* < 0.001.

To test the model we developed, we conducted mediation analysis on the quantitative data. The results of the correlation analysis using the Pearson's correlation coefficients are shown in Table 2. The results of correlation analysis showed that both normative commitment and job satisfaction were positively related to the other four dimensions of organizational commitment, while such relation was also found between normative commitment and job satisfaction. To investigate the mediating effect, a bootstrap method with 5000 bootstrap samples and 95% confidence interval (CI), including lower limit CI (LLCI) and upper limit CI (ULCI), was used. After controlling for demographic characteristics, it was demonstrated by the absence of 0 throughout the CI that affective commitment (LLCI = 0.09, ULCI = 0.28), ideal commitment (LLCI = 0.15, ULCI = 0.35), choice commitment (LLCI = – 0.09, ULCI = – 0.03), and economic commitment (LLCI = 0.05, ULCI = 0.26) have a significant indirect effect and their parallel multiple mediations on the association between normative commitment and job satisfaction was established. The mediating effect was calculated as 0.18 (affective commitment), 0.25 (ideal commitment), – 0.06 (choice commitment), and 0.15 (economic commitment). Moreover, since the direct effect of affective commitment on job satisfaction was significant (LLCI = 0.11, ULCI = 0.31), the mediators above were identified to have a part mediating effect instead of the total mediating effect (Table 3 and Figure 2).

**Table 2:** Correlation between job satisfaction and five dimensions of organizational commitment.

	**Mean**	**SD**	**NC**	**JS**	**AC**	**IC**	**EC**	**CC**
NC	19.52	3.41	1					
JS	80.27	13.58	0.75*	1				
AC	19.14	3.59	0.81*	0.75*	1			
IC	19.08	4.01	0.79*	0.79*	0.79*	1		
EC	18.53	3.84	0.78*	0.74*	0.81*	0.81*	1	
CC	14.59	4.52	0.40*	0.29*	0.50*	0.41*	0.54*	1

NC, Normative commitment; JS, job satisfaction; AC, affective commitment; IC, idea commitment; EC, economic commitment; CC, choice commitment.

^*^P < 0.001.

**Table 3:** Mediation analysis.

**Effect**	**Pathway**	**Effect value**	**95% LLCI/ULCI**
Direct effect	Normative commitment—job satisfaction	0.21	0.11/0.31
Mediating effect	Normative commitment—affective commitment—job satisfaction	0.18	0.09/0.28
	Normative commitment—ideal commitment—job satisfaction	0.25	0.15/0.35
	Normative commitment—economic commitment—job satisfaction	0.15	0.05/0.26
	Normative commitment—choice commitment—job satisfaction	−0.06	−0.09/-0.03
Total mediating effect		0.52	0.43/0.61

## Discussion

To our knowledge, this is the first study about primary care providers' job satisfaction and organizational commitment in China after massive restriction policies ended. This study found that the job satisfaction and organizational commitment of primary care providers remained high: the average scores for job satisfaction and organizational commitment were 80.6 and 90.8, respectively. The thematic analysis revealed one major theme: ethical and moral responsibility to provide care as primary care providers, on which we established a mediation model. The mediation analysis revealed that normative commitment could positively affect the other four dimensions of organizational commitment and job satisfaction. The direct effect of affective commitment on job satisfaction was significant (LLCI = 0.11, ULCI = 0.31), and the mediators identified a partial mediating effect instead of a total mediating effect.

However, due to the unpredictability of the pandemic, we do not have data about the situation before the COVID-19 breakout or during the restriction period. Considering the comparability and accessibility, we identified four possible homogenous groups from prior studies to compare to our findings. Wang surveyed Beijing's primary care providers in 2019, and the results showed that the average job satisfaction total score was 71.8 (Wang, 2020). In 2018, Jiao conducted a similar research on the general practitioner in primary care institutions in Shanghai and reported that the average job satisfaction total score was 71.5 (Jiao and Wang, 2020). Both results showed a slightly lower level of job satisfaction than ours. Liu believed that medical worker in China took full responsibility for patients' wellbeing and overcame various difficulties through their resilience and the spirit of professional dedication (Liu et al., 2020). We also observed the same experience in our research. Besides, during the restriction period, Yu assessed the job satisfaction of 455 frontline medical staff and the average score was 82.58 which was slightly higher than our results. Yu also believed that the financial and mental support from the government and gratitude from the whole society improved the professional fulfillment and job satisfaction of frontline medical staff. We guessed that primary care providers achieved self-realization by fulfilling their duty to society during the COVID-19 pandemic. Therefore, the level of job satisfaction and ideal commitment increased. However, as COVID-19 restrictions ended, the extra benefits from the government would also decrease. As people become more and more accustomed to life in the pandemic, their gratitude to medical staff may disappear. Consequently, the ending of massive restrictions was unlikely to improve the job satisfaction of primary care providers and we estimated that their job satisfaction would fall back to the pre–COVID-19 level.

With regard to organizational commitment, we identified two possibly relevant studies, one from China before the pandemic and one from Iran during the pandemic. The Iranian study showed that at the time of the coronavirus outbreak, healthcare workers had very positive and high organizational commitments (Aghalari et al., 2021). However, the authors did not mention any COVID-19 restriction information in their study and the measure tool was different from ours; therefore, we could not compare two results quantitatively. Rui's study in China showed that the average total scores for organizational commitment, affective commitment, normative commitment, ideal commitment, economic commitment, and choice commitment were 90.0, 19.3, 19.7, 17.7, 17.4, and 16.0, respectively (Rui and Huang, 2021). Although the level of organizational commitment remained stable, it seemed that the COVID-19 pandemic positively impacted ideal commitment and economic commitment, whereas it impacted choice commitment in a contrary way. We speculate that the extra subsidies from the government might have improved primary care providers' economic commitment. Regardless, before the COVID-19 breakout or during, the ideal commitment was the lowest dimension in organizational commitment. We believe one reason for the low ideal commitment was that only a small proportion (20.0% in this study) of primary care providers received budgeted posts. In other words, most primary care providers were employed as contract employees and were not permanently employed by the primary care institution. As for the decrease in choice commitment, we believe that the pandemic had made primary care providers realize the value of their professional skills, and they were less afraid of changing working institutions as the need for their skillset was in high demand. Given that the end of COVID-19 restrictions resulted in a decrease in primary care providers' income, a brief increase in resignation was expected, particularly for those without a budgeted post. However, we do not believe that the above changes are permanent. It might just be stress and stress-release reaction within health care providers, and it could disappear with the gradual social or medical end of the pandemic.

Another interesting finding from our study was the impact of organizational commitment on job satisfaction. Most studies currently focus on the relationship between organizational commitment and job satisfaction, that is, the impact of job satisfaction and its dimensions on organizational commitment or grouping them together as a factor influencing another variable. Studies about normative commitment and its impact on job satisfaction are very limited and the relation between them is still unclear. Gorgulu believed that there is a positive correlation and significant relation between normative commitment and job satisfaction (Gorgulu and Akilli, 2017). Jahangir's study in Iran got the same result (Jahangir and Shokrpour, 2009). Besides, Jahangir also found normative commitment was positively related to affective commitment (Jahangir and Shokrpour, 2009). We hypothesized a model based on qualitative interview findings that organizational commitment, in turn, affected job satisfaction, and verified it with quantitative data. The normative commitment of primary care providers positively affected the other four dimensions of organizational commitment as well as job satisfaction. The finding suggested that it was possible to improve primary care providers' job satisfaction and loyalty by helping them achieve self-realization. A possible explanation is that the roles and functions of Chinese primary care providers have been significantly emphasized by both government and society during the COVID-19 time. Actually, in the last decade, the primary care system was relatively ignored due to various reasons. Compared with care providers in the hospital, primary care providers have lower incomes, welfares, and individual development opportunities (Li et al., 2019). The waves of COVID-19 have proved that a hospital-centered system could not meet this challenge. Besides, during the restriction time, a lot of COVID-19 control and prevention works were conducted by primary care providers, as mentioned above. Therefore, a social consensus emerges that China needs a strong primary care system. Such social concern raised the morale of primary care providers and made them aware of their social values.

The results can be used to inform the policymaker on its restriction policies and help them to design interventions that can maximize job satisfaction during the COVID-19 pandemic. First, as part of our findings, we think that the end of massive social retractions would not improve the job satisfaction and organizational commitment of primary care providers. With lower choice commitment, we think the wave of quitting might be foreseeable in China, which has been witnessed in many countries (Fronda and Labrague, 2022). Second, the ideal commitment and economic commitment improved during the COVID-19 pandemic time. Therefore, we believe that budgeted posts and financial bonuses are two effective ways to maintain primary care providers' morale. Actually, the budgeted post has been the top social topic in China and a significant portion of Chinese graduates (not just medical graduate students) choose a job with budgeted posts as their priority over any other jobs. Besides, we also found that the normative commitment of primary care providers positively affected the other four dimensions of organizational commitment as well as job satisfaction. Therefore, policymakers can design interventions to maintain the normative commitment, like publicizing the importance of primary care providers to society or emphasizing the responsibility of health workers.

There still exist several limitations in the present study. First, the study utilized the cross-sectional design, therefore, cannot assume cause and effect association. Second, we only surveyed primary care providers in the urban area, namely District H, and the situation in the rural area could be different. Third, we utilized a webpage-based tool to conduct the survey and about 53.4% of all primary care providers in District H participated in the survey, which might lead to a response bias. In addition, this was not a mandatory survey, so the responders could be those who had negative feelings about their job and thus looking for changes. Furthermore, because we used a webpage-based tool, some elderly primary care providers may be unfamiliar with cellphones and thus hesitant to participate in the survey. Lastly, work-related stress has been proven associated with job satisfaction of medical staff (Khamisa et al., 2015). In this study, we did not survey the work-related stress, which may stop us from some findings. The mediation analysis is also noteworthy. Mediation analysis is prominent in psychological theory and research. During this process, a mediating variable transmits the effect of an independent variable on a dependent variable, which provides more interpretability for specific research (MacKinnon et al., 2007). One of the most difficult aspects of creating mediation analysis is determining how to create a path or casual relationship between variables. In this study, we utilized a mixed-method study design and the pathway was established based on qualitative interviews (also proved by other studies). Although a significance test is provided, some key variables may be omitted, resulting in model defaults. Therefore, the relation, especially the mechanism, on how normative commitment affects the other four dimensions of organizational commitment and job satisfaction is still unclear. More studies are needed on this topic.

## Conclusion

When most COVID-19 restriction policies ended in China, the job satisfaction of primary care providers was higher than that reported in pre–COVID-19 surveys and lower than that reported in the restriction time study. Compared with the study before the pandemic, we observed the improvement of primary care providers' ideal commitment, economic commitment, and the decline of choice commitment. We believe that the job satisfaction and organizational commitment of primary care providers will return to levels before the pandemic and during this estimated process, a brief rise in resignation is predictable.

By verifying the proposed mediation model, we think that normative commitment positively affects the other four dimensions of organizational commitment and job satisfaction for primary care providers, which suggests a possible way to motivate primary care providers when restrictions end.

## Data availability statement

The datasets presented in this article are not readily available because they are subject to ongoing research. Requests to access the datasets should be directed to the corresponding author.

## Ethics statement

Ethical review and approval was not required for the study on human participants in accordance with the local legislation and institutional requirements. Informed consent was obtained from all individual participants included in the study.

## Author contributions

QW: conceptualization, methodology, investigation, and writing—original draft. XL: data curation, and formal analysis, validation. TW: conceptualization, methodology, and investigation. ZZ, LY, SG, and HL: writing—review and editing. QS: methodology, resources, supervision, writing—review and editing. All authors contributed to the article and approved the submitted version.

## Conflict of interest

The authors declare that the research was conducted in the absence of any commercial or financial relationships that could be construed as a potential conflict of interest.

## Publisher's note

All claims expressed in this article are solely those of the authors and do not necessarily represent those of their affiliated organizations, or those of the publisher, the editors and the reviewers. Any product that may be evaluated in this article, or claim that may be made by its manufacturer, is not guaranteed or endorsed by the publisher.
